# Lumican in Carcinogenesis—Revisited

**DOI:** 10.3390/biom11091319

**Published:** 2021-09-06

**Authors:** Eirini-Maria Giatagana, Aikaterini Berdiaki, Aristidis Tsatsakis, George N. Tzanakakis, Dragana Nikitovic

**Affiliations:** 1 Laboratory of Histology-Embryology, Department of Morphology, School of Medicine, University of Crete, 71003 Heraklion, Greece; eirini_gt@hotmail.com (E.-M.G.); berdiaki@uoc.gr (A.B.); tzanakak@uoc.gr (G.N.T.); 2 Laboratory of Toxicology, Department of Morphology, School of Medicine, University of Crete, 71003 Heraklion, Greece; tsatsaka@uoc.gr

**Keywords:** lumican, cancer, extracellular matrix, proteoglycans, metastasis, cancer cell growth, motility, biomarker

## Abstract

Carcinogenesis is a multifactorial process with the input and interactions of environmental, genetic, and metabolic factors. During cancer development, a significant remodeling of the extracellular matrix (ECM) is evident. Proteoglycans (PGs), such as lumican, are glycosylated proteins that participate in the formation of the ECM and are established biological mediators. Notably, lumican is involved in cellular processes associated with tumorigeneses, such as EMT (epithelial-to-mesenchymal transition), cellular proliferation, migration, invasion, and adhesion. Furthermore, lumican is expressed in various cancer tissues and is reported to have a positive or negative correlation with tumor progression. This review focuses on significant advances achieved regardingthe role of lumican in the tumor biology. Here, the effects of lumican on cancer cell growth, invasion, motility, and metastasis are discussed, as well as the repercussions on autophagy and apoptosis. Finally, in light of the available data, novel roles for lumican as a cancer prognosis marker, chemoresistance regulator, and cancer therapy target are proposed.

## 1. Introduction-Cancer and ECM

Carcinogenesis is a multifactorial process with the input and interactions of environmental, genetic, and metabolic factors [1,2]. During this process, normal cells are transformed and exhibit enhanced survival, aggressive growth, motility, and invasion, as well as the capability to remodel their microenvironment. Indeed, the altered tumor microenvironment (TME) and the interactions therein facilitate cancer expansion [3,4]. The complex milieu of the TME, in addition to tumor cells, contains blood vessels, tissue non-malignant cells, stromal cells, infiltrating immune cells, and the modified extracellular matrix (ECM) characteristic for each phase of cancer progression [4,5]. Thus, the complex ECM structure consisting of fibrillar proteins, proteoglycans (PGs), and glycosaminoglycans (GAGs) is extensively reorganized [6] and, together with the cellular compartment, forms the new neoplastic organ [7].

Furthermore, the remodeled ECM creates a permissive environment supporting all tumor cell functions [4,8,9,10]. Importantly, ECM cues coordinate the different effectors of the TME and modulate the plethora of signaling pathways involved in the propagation of the “hallmarks of cancer” [2,11]. Moreover, the tumor ECM created by specific stromal cell subsets boosts the tumor immune escape mechanisms, triggering and sustaining an immunosuppressive networkof immunoregulatory cues [12]. In addition, tumors commonly exhibit desmoplasia, an increased deposition and cross binding of the ECM proteins where the cancer-associated fibroblasts (CAFs) as primary ECM producers are the main cell type [13].

Likewise, fibrosis, characterized by the abnormal accumulation of collagen perpetrated and supported by mechanisms including wound healing, ECM degradation, and epithelial-to-mesenchymal transition (EMT), significantly impacts cancer progression and putative therapeutical strategies [14]. Indeed, the resulting ECM “stiffening” is a vital regulator of tumor cell functions.

Therefore, both the cellular and ECM components of the formed neoplastic “organ” are extensively modified during tumorigenesis and regulate cancer progression as previously discussed [1,8,15].

## 2. SLRPs Structure and Function—Focus on Lumican

PGs are glycosylated proteins that participate in the formation of the ECM. These hybrid molecules consist of a protein core into which one or more GAG chains are covalently bound. Four different types of GAGs can be attached to PGs’protein core, heparan sulfate (HS), chondroitin sulfate/dermatan sulfate (CS/DS), and keratan sulfate (KS) chains.

Forty-five PGs have been identified up until now, and they exhibit extensive variability in their protein core composition and glycosylation pattern. Three criteria are considered when classifying PGs: their topology (cellular or subcellular), overall gene/protein homology, and the presence of specific protein modules within their respective protein cores [16].

Specific structural features determine PG functions, such as the core protein structure, GAG chains’ composition, and sulfation pattern [17]. Indeed, their protein cores characterize the existence of unique protein modules thatmembers of a given class often share, such as the PDZ-like, laminin-like, and EGF-like domains [16]. Furthermore, it has been shown that specific sulphation motif sequences within the CS/HS chainscarry biological information to the cells [17,18]. Notably, the binding partners for GAG chains remain partly uncharacterized, and different PG classes seem to function by utilizing overlapping signaling with various outcomes [17].

Although PGs play an essential role in regulating cellular processes like tissue homeostasis and development [19], their expression pattern and functions are changed during tumorigenesis and are correlated with cancer development and progression [1,8]. Thus, solid tumors’ behavior and differentiation status are closely associated with altered PG expression profiles, with epithelial tumors exhibiting a more discrete PG phenotype than mesenchymal tumors [20].

The small leucine-rich proteoglycans (SLRPs) are a distinct family of 18 proteins with unique characteristics. They consist of a small protein core (36–42 kDa) with a variable number of central leucine-rich repeats (LRRs) variously substituted with GAG chains [2,16]. LRRs exhibit different amino acid sequences in discrete SLRPs, their size varying between 20 and 29 residues, while the N and C-terminal regions of the protein core bear numerous cysteine residues [21,22].

SLRP classification is based on the conservation of the amino acid residues of the protein core, the organization of disulfide bonds at the molecule’s N- and C-terminal regions, and their gene/protein homology. They are categorized into five different classes [23]. PGs belonging to classes I, II, and III are canonical, while classes IV and V are non-canonical [24]. Class I SLRPs, like biglycan and decorin, are mainly substituted with CS/DS chains; class II SLRPs, like lumican (LUM), are covalently bound with KS chains. In contrast, class III members can bear KS chains (osteoglycin), CS/DS chains (epiphycan), or do not carry GAG chains (opticin) like classes IV and V SLRPs [16,25].

Many studies have shown that SLRPs interact with diverse cell membrane receptors, cytokines, chemokines, and ECM molecules [16,26]. Notably, most SLRP family members undergo different post-translational glycosylation [27] and are competent to regulate signal transduction mechanisms, and affect various cellular functions, like proliferation, migration, and differentiation [2,28]. In addition, many studies have also reported that SLRPs’ interaction with growth factors or tyrosine kinase receptors affects cellular behavior and tumor progression [29,30,31,32].

## 3. Lumican Structure, Function, and Expression-Correlation with Carcinogenesis

Lumican, a class II SLRP, has a 38 kDa protein core exhibiting four distinct regions: a 16 amino acid peptide, a negatively charged N-terminal region containing tyrosine sulfate and disulfide bonds, a 6–10 LRR motifs characterized by common molecular architecture that supports protein interactions, and a C-terminal region consisting of two conserved cysteine residues [22]. Amino acid sequencing data revealed the presence of four possible substitution positions with KS chains or oligosaccharides within the LRR region [33]. However, it seems that not all of these positions can be used for glycosylation of the protein by KS chains [34]. Moreover, it has been suggested that there is an increase in non-glycosylated forms of lumican with age due to the decrease of KS synthesis [35].

The lumican gene is located on chromosome 12q21.3-q22 [33], and its expression is significantly altered between tissues during different developmental stages. For example, its expression is early detectable in the chicken cornea during fetal development [36]. Still, it is not expressed until birth in human cartilage [37], indicating species-dependent roles of lumicanduring embryogenesis.

Lumican participates in the structural organization of tissues. Thus, lumican-deficient mice collagen fibrils exhibit an increased diameter forming a disorganized matrix [38]. Early studies in the mouse model showed that lumican is widely distributed in most interstitial connective tissues [39]. Indeed, this SLRP is an important PG of the bone matrix, and its’ expression is positively correlated to the bone differentiation stage [40]. Furthermore, lumican is highly expressed in the skin and cornea [41,42], where lumicandeficiency is translated into tissue disfiguration with resulting skin laxity and a decrease in corneal clarity [41,42]. Lumican expression in parenchymal cells such as urothelial and colon epithelium, albeit at lower levels, has been determined [43,44]. The role of lumican, however, partly overlaps that of fibromodulin [40]. Indeed, these two class II PGs are extensively expressed in collagenous connective tissues where they significantly affect tissue integrity [45].

Notably, lumican is involved in cellular processes associated with tumorigeneses, such as EMT (epithelial-to-mesenchymal transition), cellular proliferation, migration, invasion, and adhesion [32,46,47]. Furthermore, lumican is expressed in various cancer tissues and is reported to have a positive or negative correlation with tumor progression [26]. More specifically, immunohistochemistry demonstrated a much higher expression of lumican in cancerous gastric tissues than normal tissues.In this cancer type, the lumican expression was correlated with histological classification, cancer dissemination to secondary sites, and lymphatic metastasis [48]. Furthermore, the TCGA database analysis showed a higher expression of lumican in the gastric cancer tissues than the neighboring non-tumor tissues [49]. This was correlated, as probed by the Kaplan−Meier analysis, with a poor prognosis. Moreover, a multivariate analysis demonstrated a strong positive association between a high LUM expression and poor overall survival. Notably, lumican enhanced 14 signaling pathwayspotentially correlated with this cancer progression [49]. On the other hand, the expression of lumican and versican by cancer-associated fibroblastswas associated with a poor relapse-free and overall survival of esophageal squamous cell carcinoma [50].

In colon cancer, the lumican expression was correlated with lymph node metastasis and a lower survival rate [51]. Specifically, lumican was detected in the cytoplasm of cancer cells in 62.7% of 158 patients undergoing curative surgery for advanced colorectal cancer with lymph node metastasis. Notably, lumican expression was positively associated with the spread of lymph node metastasis and had lower survival rates [51]. This study is in accordance with the UALCAN database analysis, which determined a high lumican mRNA expression in colorectal adenocarcinoma tissues [52]. The application of the univariate and multivariate COX analysis and Kaplan−Meier method to this dataset identified the lumican expression as a poor prognosis marker.

Moreover, LinkedOmics demonstrated that the LUM expression was strongly associated with miR200 family expression and tumor immune escape. Indeed, it was determined that lumican facilitated colon cancer progression through a miRNA200-dependent epithelial-to-mesenchymal progression. Zang et al. suggested that lumican is a potential target in colon cancer [52]. Furthermore, when the tissue microarrays and tissue sections were analyzed, lumican was found to be expressed by both transformed cells and the stroma of colon adenomas and carcinomas. Notably, it was more frequently detected in carcinoma than adenoma cells and in carcinomas and high-risk adenomas combined compared withlow-risk adenomas [44]. On the other hand, the lumican expression by the colon cancer cells was positively correlated with a longer disease-specific and disease-free survival in stage II colon cancer patients, and a more prolonged disease-specific survival in microsatellite-stable stage II colon cancer patients, suggesting a disease stage dependence [53].

In pancreatic cancer, the expression of lumican was demonstrated by a immunohistological analysis [54], where pancreatic stellate cells were identified as a major source of this PG [55]. Notably, a small fraction of the PDAC tumor mass is attributed to cancer cells, the majority consisting of desmoplastic TME with abundant activated fibroblasts, leukocytes, and pancreatic stellate cells [56].

Melanoma cells do not express lumican, but the increased expression of lumican to the peritumoral stroma is negatively correlated withthis tumor growth [57]. The correlation of the lumican expression and various tumor progression is summarized in Table 1.

### Lumican Regulates Cancer Cell Growth, Invasion, and Metastasis

Many studies have shown that lumican modulates tumor cells’ proliferation, invasion, and metastasis with different mechanisms, either enhancing or preventing cancer progression. A characteristic example is the regulation of the growth factor activity in mesenchymal tumors and the effects on these cancer cell functions [32,46,58]. Lumican is the most abundant SLRP produced by HTB94 human chondrosarcoma cells and a positive regulator of these cells’ growth. Indeed, lumican deficiency significantly inhibits basal and IGF-I induced HTB94 cell growth. The oncogenic action of IGF-I is mediated by its receptor, IGF-IR, whose phosphorylation levels are strongly attenuated in lumican-deficient HTB94 cells. Furthermore, lumican affects ERK1/2 activation, which seems crucial to IGF-I-dependent HTB94 cell growth [32].

Likewise, lumican expression and secretion by osteosarcoma Saos-2 and MG63 cells are correlated with their differentiation [46]. Indeed, the well-differentiated Saos-2 cells had a negative growth response to lumican, while their migration and the chemotactic response to fibronectin were enhanced. Moreover, the mechanism was mediated by Smad-2 downstream signaling. On the other hand, these cellular functions of poorly differentiated MG63 cells are not affected by low endogenous lumican levels [46]. Further studies revealed that lumican-deficient Saos-2 cells exhibited increased adhesion onto fibronectin, which was abolished upon neutralization of the endogenous transforming growth factor β2 (TGF-β2) activity. On the other hand, treatment with exogenous TGF-β2 was shown to stimulate Saos-2 cell fibronectin-dependent adhesion [58]. Nikitovic et al. thus suggested that lumican is an upstream regulator of the TGF-β2/Smad 2 signaling pathway in an osteosarcoma cell model.

Lumican pro-tumorigenic effects are also observed in gastric, bladder, colon, clear cell renal, and liver cancers [59,60,61,62]. A high lumican expression in gastric cancer tissues indicates a poor patient prognosis [48]. Indeed, Wang et al. showed that the increased expression of lumican by human gastric cancer-associated fibroblasts is positively associated with lymph node metastasis, TNM stage, depth of invasion, and a poor survival rate of gastric cancer. Indeed, lumican promotes gastric cancer cell growth by activating the integrin β1/FAK signaling axis [63].

In human colon adenocarcinoma cells, lumican overexpression was found to be accompanied by changes in the actin polymerization state, immediately associated with cancer cells migration and higher metastatic potential [61,64]. In addition, hepatic cancer HepG2 and MHCC97H cells express more lumican in comparison with normal Lo02 hepatocytes. Transfection of hepatic cancer cells with shRNAs specific for lumican resulted in decreased invasion and migration mediated by reducing the ERK-1 and JNK activation status [65].

In a neuroblastoma model, lumican was a downstream mediator of FOXO3 transcription factor action and enhanced these cells’ migration. FOXO3 is correlated with a poor outcome in high-stage neuroblastoma due to its’ chemoprotective and angiogenesis-stimulating properties [62]. Notably, upon inhibiting FOXO3 by the small molecular weight compound repaglinide, the binding of FOXO3 to the LUM promoter was attenuated, abrogating the FOXO3-dependent lumican expression and decreasing neuroblastoma cell 2D- and 3D-migration [62]. Regarding clear renal cell carcinoma (cRCC), the microarray analysis demonstrated a higher expression of matrix regulators lumican and CEACAM6 in metastatic tissues than patient-matched primary tissues [60]. Indeed, these authors conclude that the ECM genes a re crucial triggers resulting in visceral, bone, and soft tissue metastases in cRCC.

**Figure 1:**
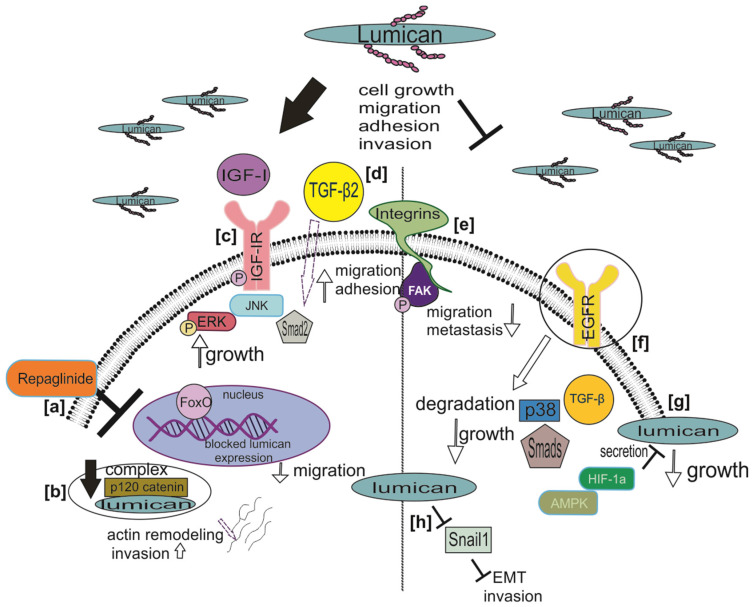
Schematic representation of lumican’s signaling in carcinogenesis. (**a**) Inhibiting the binding of FOXO3 to the lumican promoter by the small molecular weight leads to decreased FOXO3-dependent lumican expression and neuroblastoma cell migration. (**b**) Upon lumican downregulation, its colocalization with p120 catenin (p120ctn) decreases, leading to actin cytoskeleton remodeling and accelerated lung cancer cell invasion. (**c**) Lumican-deficient hepatic cancer cells show decreased invasion and migration mediated by reducing IGF-IR, ERK-1, and JNK activation status. (**d**) Lumican is an upstream regulator of the TGF-β2/Smad 2 signaling pathway in an osteosarcoma cell model, regulating cell adhesion. (**e**) Lumican interacts with the integrin β1/FAK signaling axis, affecting tumor progression positively or negatively. (**f**) Lumican induces the dimerization of the EGFR receptors and their subsequent uptake and degradation, leading to attenuated PDAC cell growth. (**g**) Hypoxia significantly reduces lumican secretion from pancreatic stellate cells and results in attenuated PDAC cell growth. (**h**) Lumican affects the signaling of Snail, an EMT trigger molecule that facilitates cancer metastasis, attenuating melanoma metastasis to the lungs.

On the other hand, the lumican expression is suggested to attenuate discrete tumor progression, including pancreatic cancerand melanoma, as recently discussed [59]. Thus, lumican was shown to inhibit cancer cell proliferation inthe early stages of pancreatic ductal adenocarcinoma (PDAC) [58]. Indeed, it was demonstrated that exogenous lumican induces features of a quiescent state, including growth arrest, apoptosis, and chemoresistance [66]. Interestingly, this was partly executed through an EGFR-dependent mechanism, as lumican induced the dimerization of the EGFR receptors and the subsequent uptake and degradation [66]. Indeed, the interactions of lumican with growth factors/growth factor receptors and the effects on tumor cell functions are schematically depicted in Figure 1. A study with patient tumor tissues, ex-vivo cultures of patient-derived xenografts (PDX), pancreatic ductal adenocarcinoma (PDAC) stellate, and tumor cells was conducted to investigate whether hypoxia within the tumor microenvironment alters stromal lumican expression and secretion [67]. Li et al. demonstrated that hypoxia significantly reduced lumican secretion from pancreatic stellate cells and induced autophagy in these cells, as well as in ex vivo cultures of PDX, but not cancer cells cultured under 2D conditions [67].

Regarding melanoma, lumican also seems to be negatively correlated with its progression. In vivo experiments in lumican-null mice revealed that lumican is an endogenous inhibitor of melanoma growth and modulates the response to TAX2, an anticancer cyclic peptide. Notably, the null mice tumors were twice as large as the wild-type animal tumors [68]. Furthermore, the lumican protein core was shown to inhibit melanoma cells’ migration. Indeed, lumican induced changes in actin filaments and β1 integrin ligation, and enhanced vinculin accumulation in the cell cytoplasm, destabilizing focal adhesion complexes. In addition, the phosphorylation levels of FAK were significantly decreased. Combining these alterations in the cytoskeleton and the adhesion molecules’ activation status may contribute to the lumican anticancer effect in A375 melanoma [69].

Moreover, lumican was shown to affect the signaling of Snail, the main EMT trigger, cancer-facilitating molecules.Thus, when the Snail1 overexpressing B16F1 melanoma cells and the Mock-B16F1 cells were inoculated in Lum^+/+^ and Lum^−/−^ mice, a significantly higher number of metastatic nodes were detected in the lungs of Lum^−/−^ mice inoculated with Snail-overexpressing B16F1 cells.These data suggest that endogenous lumican of the wild-type mice markedly attenuates melanoma metastasis to the lungs. Notably, the expression and activities of molecules, including ECM mediators, correlated to the invasive phenotype were altered in in vitro models [70]. Another study, in an immunocompetent model of melanoma, implanted in Lum^−/−^ vs. wild type syngeneic mice, concluded that endogenous lumican modulates the organization of the tumor matrix regarding the intratumoral distribution of matrix proteins, growth factors, and stromal cells in a manner correlated with disease progression [68].

Furthermore, lumican attenuated the growth of melanoma cells and downregulated the response to the anticancer validated peptide TAX2. Indeed, Jeanne et al. identified lumican as an essential regulator of the tumor matrix structure and function [68]. Recently, in a mouse model of primary melanoma, the lumican-derived L9Mc peptide abrogated the growth and increased the apoptosis of B16F1 cells, as determined by infrared spectral imaging and histopathology [71].

The transcription factor FOXO3 is associated with a poor outcome in high-stage neuroblastoma (NB), facilitating chemoprotection and tumor angiogenesis. In addition, FOXO3 stimulates metastasis formation in other tumor entities, one of the biggest challenges in treating aggressive NB. The SLRP member lumican has been determined as a FOXO3-regulated gene that stimulates cellular migration in NB [62].

For some cancer types, such as lung cancer, contrasting roles of lumican were reported. Non-small lung cancer cell lines growth is negatively impaired by lumican, as lumican-deficient H460 and A549 cells exhibit a prolonged doubling time and retarded growth. Specifically, lumican deficiency affected central spindle and midbody formation, resulting in chromosome missegregation, multinucleated cells, increased chromosome instability, and retarded cell growth [72]. In contrast, a separate study reported that the depletion of lumican increased lung cancer cell invasion. Upon lumican downregulation, its colocalization with p120 catenin (p120ctn), an intracellular scaffolding protein of the catenin family, is decreased, leading to morphological changes and actin cytoskeleton remodeling, which accelerated cell invasion [73].

Tumor aggressiveness is connected to EMT, as the differentiation state of cancer cells defines their invasive properties [74]. A recent study in breast cancer in vitro showed that lumican treatment in combination with the knockdown of ERα and the suppression of ERβ can regulate these cells’ differentiation state, morphology, expression of matrix effectors, and cell behavior [75]. Indeed, the effects of lumican seem to be hormone-receptor dependent as the aggressive metastatic ERβ-positive MDA-MB-231, the ERβ-suppressed (shERβMDA-MB-231) cells, and the ERα-positive MCF-7/c breast cancer cells of a low metastatic ability exhibit varying responses to lumican. Thus, exogenous lumican increases the expression of α2 and β1 integrins in MDA-MB-231 and in shERβMDA-MB-231 compared withMCF-7/c cells. Furthermore, specific integrin-dependent downstream signaling pathways, including FAK, ERK 1/2 MAPK 42/44, and Akt, were attenuated by lumican [76]. Moreover, Karamanou et al. suggested that treating breast cancer cells seeded to 3D collagen cultures with lumican enhancedcell−cell contacts and cell grouping, initiating a less invasive phenotype [47]. A separate study showed that this SLRP might inhibit or even reverse the metastatic features that breast cancer cells acquire undergoing EMT by increasing the gene expression of the EMT inhibitor miR-200b [70]. On the other hand, Leygue et al. showed that lumican expression differs during breast tumorigenesis, andlumican mRNA, identified in the tumor stroma, is correlated with a higher tumor grade and lower expression of estrogen receptors and younger age of the patients [77].

Lumican effects on different cellular functions of cancer cells are summarized in Table 2.

The multifaceted signaling roles of lumican in carcinogenesis are schematically depicted in Figure 1.

## 4. Lumican Modulates Cancer Cell Motility

The mechanisms presented in this section involve the interplay of lumican with specific cell membrane receptors, which leads to the activation of downstream signaling pathways. A crucial downstream mediator is focal adhesion kinase (FAK), which participates in focal adhesion turnover, actin cytoskeleton reorganization, and MMP expression, and regulates cell motility and, therefore, metastasis. One of the key examples is the role of lumican on melanoma cell adhesion and motility. Initially, the lumican protein core was shown to inhibit melanoma cells’ migration. Indeed, lumican induced changes in actin filaments and β1 integrin ligation, and enhanced vinculin accumulation in the cell cytoplasm, destabilizing focal adhesion complexes. In addition, the phosphorylation levels of FAK were significantly decreased. Combining these alterations in the cytoskeleton and adhesion molecules’ activation status is suggested to contribute to the lumican anticancer effect in A375melanoma [69].

Moreover, the potential anti-metastatic role of lumican in melanoma by inhibiting the membrane-type matrix metalloproteinase (MMP)-14 activity and melanoma cell migration in vitro has been studied in vitro and in vivo [78,79,80,81,82]. MMP-14 is necessary for cell migration, because it modulates the activity and expression of downstream MMPs; activates integrins and CD44 [73]; and regulates intracellular signaling involving MAPK, FAK, Src, and Rac [69,76,83,84,85,86]. Importantly, the glycosylated full-length lumican was likewise shown to block the MMP-14 activity, behaving as a competitive inhibitor [79]. Indeed, lumican inhibits the degradation of ECM by inhibiting MMP-14, then influencing integrin clustering, modulating focal adhesion site stability and FAK phosphorylation at Tyr-397, leading to the inhibition of melanoma cell migration [81]. Moreover, the lumcorin peptide corresponding to a sequence of 17 amino acids carried by the core protein of lumican inhibits melanoma cell chemotaxis in a manner similar to lumican protein [87]. Interestingly, lumcorin triggered the expression of an intermediate form of MMP-14 (~59 kDa) and attenuated its activity [88].

During EMT, where cancer, including melanoma cells, acquire enhanced motility, vital participation of Snail signaling has been shown [89]. Notably, lumican attenuated the Snail-induced MMP-14 activity and migration in B16F1, but not in HT-29 cells. In Snail overexpressing Snail-B16F1 cells, lumican significantly inhibits and melanoma primary tumor development. Thus, a lumican-based strategy targeting the Snail-induced MMP-14 activity might be helpful for melanoma treatment [89]. Lumican actions involving processes like reduced formations of cytoskeletal projections such as lamellipodia and invadopodia were also associated with decreased ZO-1, keratin 8/18, integrin β1, and MT1-MMP expression/activity [90].

Indeed, lumican can affect the biological roles of various downstream mediators, including integrins, cyclin D1, cortactin, vinculin, hyaluronan synthase 2, heparanase, and the phosphorylation of AKT, p130 Cas, and GSK3α/β [70,79,80,88,91,92,93].

Other cancer cell types that have been studied regarding the lumican-dependent motility effects include lung, breast, colon, liver, bladder, and pancreatic cancer, as well as neuroblastomas [3,61,62,65,69,73,79,80,88,94,95].

Thus, it has been determined that type I collagen promotes the most robust adhesion and migration of eight pancreatic cancer cell lines, explicitly mediated by the alpha2beta1integrin [94]. In continuation, Zeltz et al. determined that lumican is a specific inhibitor of alpha2beta1 integrin, attenuating the ability of A375 melanoma cells to migrate. This effect was verified in a study on Chinese hamster ovary (CHO) cells expressing the α2 integrin subunit (CHO-A2), whose ability to migrate was attenuated by lumican in contrast with the wild-type CHO cells (CHO-WT) lacking this subunit. Moreover, in the presence of recombinant lumican, the pFAK/FAK ration was strongly downregulated in CHO-A2 cells [92]. Likewise, in breast cancer, lumican significantly downregulates the migratory abilities of tumor cells in a mannerdependent on their hormone receptor status [76].

On the other hand, it was shown that lumican enhanced the adhesion and migration on the collagen of both pancreatic cancer cells and pancreatic stellate cells in a manner dependent on TGF-Β [55]. Yang et al. indicated an interplay between lumican and microtubules that acts as a molecular switch to coordinate the balance between cell adhesion and migration. Indeed, it is suggested that lumican propagates these effects through p120-catenin signaling and cytoskeletal remodeling, as well as the activities of Rac and Rho [73].

Downregulation of the lumican expression attenuated lung osteotropic cancer cell’s adhesion to various ECM components, ultimately decreasing these cells’ migration. On the other hand, the introduction of exogenous lumican restored the motility of lumican knockdown cells and enhanced the invasion of lung cancer cells in the bone niche [3].

In liver cancer cells, silencing lumican by shRNA reduced cell invasion and migration via inhibiting the activation of the ERK1/JNK pathway, suggesting that lumican is a positive regulator of these cells’ migratory abilities [65]. In the neuroblastoma, the silencing of the lumican gene in FOXO3 expressing IMR32 and SK-N-SH neuroblastoma cells or adding a FOXO3 inhibitor that restricted lumican transcription resulted in these cells’ reduced migration capacity [62]. These results suggest that FOXO3 is a lumican biological partner that is important to neuroblastoma development. Likewise, a recent study on bladder cancer showed that lumicans’ expression was more prominent in bladder cancer tissue and cell lines than in healthy adjunct tissues. Moreover, in in vitro models, the downregulation of lumican decreased bladder cancer cells’ migration by attenuating the downstream MAPK signaling [43].

The motility of colon cancer cells is also upregulated by lumican. Thus, Radwanska et al. showed that human LS180 colon cancer cells that overexpress lumican tend to create podosome-like structures. This was noted due to the redistribution of vinculin and its simultaneous colocalization with actin and gelsolin in the cells’ submembrane region [61]. Therefore, these authors conclude that the secreted lumican enhances LS180 cells’ motility. Likewise, lumican upregulates gastric cancer cell migration through an integrin β1-FAK downstream signaling pathway, as depicted in Figure 1 [63].

In summary, the effect of lumican on cancer cell motility seems to be cancer-type dependent, as both positive [3,43,55,61,62,63,68,73], negative [69,76,78,79,80,81,89,92,94], or no effect has been determined [32]. Therefore, an in-depth study of the utilized mechanisms is imminent in order to identify the genotypic phenotype of lumican-responsive cancer to develop target therapeutic strategies.

## 5. Lumican at the Crossroad between Apoptosis and Autophagy

The suppression of apoptosis together with deregulated cell growth provides two essential criteria for cancer progression [95]. Early studies have demonstrated the ability of lumican to regulate the apoptosis of corneal and embryonic fibroblasts [96]. Lumicans’ mechanism of action incorporates Fas-FasL signaling and the modulation of cell growth and apoptosis mediators, including p21 and p53 [96]. In continuation, the effects of lumican on cancer cell apoptosis were determined. Thus, B16F1 cells transfected to overexpress lumican present an initiation and/or increase ofapoptosis [97]. Moreover, in a mouse model of B16F1 melanoma primary tumor growth, lumican treatment with the L9Mc peptide increased cancer cell apoptosis [71].

In a separate cancer model, lumican secreted by stromal cells was shown to attenuate the expression and activity of hypoxia-inducible factor-1α (HIF1α)via Akt signaling, leading to the enhanced apoptosis of pancreatic cancer cells [54]. Furthermore, lumican was determined to trigger, characterized with apoptosis, a quiescent pancreatic cancer state [66]. Moreover, lumican was shown to facilitate endothelial cell apoptosis through Fas-dependent signaling. Thus, lumican-overexpressing murine fibrosarcoma (MCA102) and pancreatic adenocarcinoma (Pan02) cells providesmaller tumors in vivo compared withwild-type cancer cells [98]. This was correlated withattenuated neoplasm tissue vascular density. Therefore, lumican repressed tumor growth in this model, increasing endothelial cell apoptosis [98]. Likewise, the intensity of VEGF immunostaining and the abundance of blood vessels in melanoma lung metastasis nodules were decreased in lumican-expressing tumors. Therefore, in addition to inducing the apoptosis of melanoma cells, lumican inhibited tumor-associated angiogenesis [69]. Furthermore, it has been suggested that lumican inhibits angiogenesis through MMP14 and integrin α2β1 signaling.

Autophagy is an ancient catabolic process in which cells sequester damaged organelles and protein aggregates to process their degradation [99]. Indeed, under normal conditions, autophagy is a beneficial process dynamically regulated by starvation and other stresses [99]. However, autophagy can facilitate the viability and chemoresistance of cancer cells, the maintenance of cancer stem cells, and, in a context-dependent manner, have an inhibiting effect on tumor growth [100]. Thus, enhancing autophagy may attenuate inflammatory responses that support carcinogenesis and abrogate tumor escape from the host immune system defense mechanisms [101]. On the other hand, the upregulation of autophagy may have a pro-survival effect on cancer cells [102].

The cancer-microenvironment niche and its components, such as proteoglycans, decidedly regulate autophagy [2]. To date, a primarily an anti-autophagic role has been attributed to the lumican [103]. Indeed, chemotherapeutic agents increase the secretion of lumican in PDAC, which, by inhibiting autophagy, enhances chemotherapy-induced growth inhibition. Indeed, this effect of lumican was verified in both in vitro and in vivo PDAC models, including patient-derived xenografts [104]. However, a feedback mechanism seems to be emerging, as it was recently demonstrated that hypoxia induces autophagy in pancreatic cancer stellate cells, accomplished through an AKMP/TOR/p70S6K/4EBP signaling pathway-mediated protein degradation and synthesis inhibition [67]. Furthermore, the mechanism strongly downregulates lumican secretion through post-transcriptional regulation of pancreatic stellate cells, not cancer cells [67]. Notably, pancreatic stellate cells exhibit significant regulatory roles in tumor immunology, paracrine signaling, and metabolism in pancreatic ductal carcinoma [105].

## 6. Implications of Lumican in Cancer-Associated Inflammation

The process of tumorigenesis is intimately correlated with chronic inflammation, with a significant 20% of cancer incidences directly related to chronic infections [106]. All tumor types separately of etiology specifically interact with the immune system at all stages of carcinogenesis [107]. The ECM components play a significant role in these interactions. Indeed, the tumor microenvironment, extensive remodeling modulates the immune response [108]. Other SLRPs such as biglycan have been implicated in cancer-associated inflammation [109,110].

The available knowledge on the role of lumican in the processes of tumor-associated inflammation is restricted [26]. Some research, however, indicates a connection between lumicans’ biological effects and inflammation. Thus, for example, when using a mouse colitis model, Lohr et al. showed that lumican exacerbates the immune and inflammatory responses [111]. Specifically, Lum^−/−^ mice had a decreased secretion of cytokines such as tumor necrosis factor-alpha (TNF-α); CXCL1 secretion; correlated to the retarded translocation of and NF-κB translocation to the nucleus; and attenuated neutrophil infiltration [111]. In contrast, the Lum^−/−^ mice presented substantial weight loss and extended tissue damage compare withthe wild-type mice. These authors suggest that lumican supports the homeostasis of the intestine by facilitating the inflammatory response found to be beneficial to the initial stages of colitis [111]. Similar results were obtained in the LPS-induced sepsis murine Lum^−/−^ model. Indeed, lumican-deficient mice had a strongly downregulated inflammatory response to sterile inflammation. This was evident as an attenuated secretion of pro-inflammatory mediators, including TNF-α, IL-6,and IL-1β cytokines [112]. The correlation of lumican action to TLR signaling has been, likewise, implicated in a murine pathogen-induced inflammatory response. Thus, in mice, lumican seems to facilitate the bindingof bacteria to CD14 and the subsequent presentation of the comp0lex to TLR4 [113]. Indeed, CD-14 is a glycosyl phosphatidyl inositol-linked membrane protein that enhances TLR-2 and TLR-4 downstream signaling [114]. In this manner, lumican upregulatedbacteria phagocytosis [113]. Therefore, lumican has been characterized as a promotor of TLR4- and CD14-dependent pathogen sensing [115]. Likewise, lumican was demonstrated to modulate peripheral monocyte extravasation via Fas–FasL signaling [116]. Therefore, it seems feasible that the mechanisms mentioned above of lumican action can participate in the tumor inflammatory milieu.

## 7. Lumican as Prognosis Marker, Chemoresistance Regulator, and Cancer Target

The expression of lumican has been correlated with prognosis and disease stage in various cancer types. For example, in gastric and colon cancer, lumican expression was associated with cancer dissemination to secondary sites, lymphatic metastasis, and a poor overall survival of patients [48,51]. Moreover, in colon cancer, the expression of lumican was positively correlated with the disease stage [52]. In RCC, lumican exhibited a higher expression of lumican in metastatic tissue than patient-matched primary tissues, and is suggested as a metastases marker [60].

In melanoma and breast cancer, lumican exerts anticancer properties, and lumican-based therapeutic strategies have been examined [69,70].

Notably, lumican is suggested to modulate the response to chemotherapeutic agents. Thus, chemotherapeutic agents increased the secretion of lumican in PDAC cells, which was correlated with the extent of the therapy response. In various PDAC models, including cell lines, patient-derived xenografts, and lumican knockout mice, lumican was found to enhance the anticancer chemotherapy effect [104]. Specifically, chemotherapeutic agents in PDAC cells facilitate autophagosome formation and enhance LC3 expression through the ROS-mediated AMP-activated kinase (AMPK) signaling pathway. Conversely, lumican attenuates AMPK activity, abrogating the protective mechanism of chemotherapy-induced autophagy in in vitro and in vivo PDAC models [104]. This was correlatedwithDNA damage, apoptosis, downregulated cell viability, lactate production, glucose consumption, and release of vascular endothelial growth factor [104].

In rectal cancer patients treated with radiotherapy, apoptosis inducers (lumican, thrombospondin 2, and galectin-1) exhibited a higher expression in responders than in non-responding patients [117]. Therefore, gene expression profiling may benefit from radiotherapy response predictionand provide insights into developing novel therapeutic targets for rectal cancer.

Notably, leukemia stem cells (LSCs) have been correlated withtherapeutic failure and the relapse of acute lymphoblastic leukemia. The interaction between LSCs and bone marrow mesenchymal stem cells (BM-MSCs) results in a decreased expression of lumican by BM-MSCs cells. Importantly, a downregulated lumican expression results in attenuated apoptosis and enhanced chemoresistance to VP-16 in human pre-B cell leukemia Nalm-6 cells [118]. Therefore, reduced lumican expression by cells BM-MSCs may facilitate cancer cells’ escape from chemotherapy and immune surveillance and support leukemia relapse [119].

Based on the available data and in a cancer-type specific manner, lumican roles as anticancer agents have been proposed. Thus, as lumican may attenuate or even annulate specific EMT-correlated metastatic features in breast cancer cells, a lumican-based anticancer therapy targeting EMT could be beneficial [47]. Furthermore, as lumican modulates the response to the matrix-targeting therapy peptide TAX inhibiting tumor growth, it presents a feasible therapeutic option for neuroblastoma [68].Indeed, the attenuation of the FOXO3/LUM axis by the small molecular weight compound repaglinide is suggested as a novel strategy for neuroblastoma and other FOXO3-dependent neoplasms [62]. The latter is depicted in Figure 1.

## 8. Conclusions

**Figure 2:**
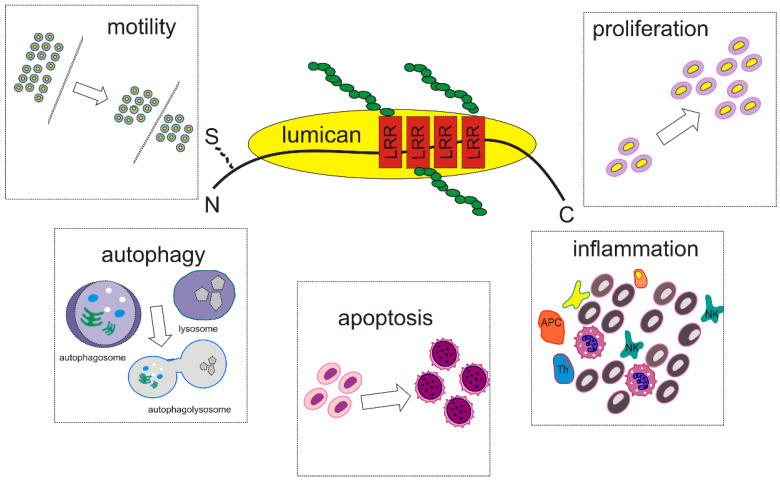
Lumican affects cancer cell behavior.Lumican alters cancer cell proliferation, migration, adhesion, invasion, metastasis, and apoptosis, and affects autophagy and inflammation signaling pathways with different mechanisms.

In conclusion, lumican is an important mediator of tumorigenesis and cancer progression involving the cellular functions of proliferation, motility, apoptosis, autophagy, and angiogenesis regulation, as represented in Figure 2. Lumican has been characterized as both an anticancer molecule and a tumor promoter. However, recent research efforts have shed more light on the characterization of its roles, which seem to depend on the tumor origin and type and disease stage.Therefore, lumican has been proposed as both a therapy target and anticancer agent. Moreover, by modulating specific biological functions, lumican can affect the response to chemotherapy and predict the response to radiotherapy. Considering that the tumor microenvironment is a complex network of different cell types, ECM components, and signaling molecules, with the ability to modulate cell growth and metastasis, defining its critical modulators is essential. The novel findings on the multivalent roles of lumicans have the potential to be translated into effective therapeutic strategies, and thus it is necessary to continue research efforts in this direction.

## Figures and Tables

**Table 1:** Lumican expression in tumor tissues and correlation to carcinogenesis.

Cancer Type	Detected Expression (Protein/mRNA)	Level of Expression	Clinical Correlation	Ref.
Gastric cancer	Protein	Overexpressed in cancerous gastric tissues compared to normal tissues	Cancer dissemination to secondary sites and lymphatic metastasis	[48]
Gastric cancer	mRNA	Higher expression of lumican in the gastric cancer tissues than neighboring non-tumor tissues	Poor overall survival	[49]
Colon cancer	Protein	Overexpressed by cancer cells	Lymph node metastasis and a lower survival rate	[51]
Colon cancer	mRNA	Overexpressed	Poor prognosis	[52]
Adenoma to colon cancer transition	Protein	Increased expression during the transition process	Cancer stage	[44]
Colon cancer	Protein	Overexpressed	Positively correlated to a longer disease-specific and disease-free survival in stage II colon cancer patients and a more prolonged disease-specific survival in microsatellite-stable stage II colon cancer patients	[53]
Pancreatic ductal adenocarcinoma (PDAC)	Protein	Overexpressed	Associated with prolonged survival after surgery	[54]
Melanoma	Protein	Not expressed by tumor cells, expressed at peritumoral stroma	Negatively associated with melanoma growth	[57]

**Table 2:** Lumican’s role in various cancer types and the mechanism of action.

	Cancer Type	Model	Alterations in Signaling Pathways	Effect on Cell Function	Ref.
Tumorigenic action	Chondrosarcoma	HTB94 human cell line (in vitro)	IGF-I/IGF-IR/ERK1/2	Cell growth	[32]
	Osteosarcoma	Saos-2 human cell line (in vitro)	TGF-β2/Smad2	Migration and adhesion to fibronectin substrate	[46,58]
	Gastric cancer	MKN45 human cell line, primary cell cultures, tissue biopsies (in vitro), and ice model (in vivo)	Integrin-β1/FAK	Cell growth, migration, and invasion	[63]
	Liver cancer	HepG2 and MHCC97H human cell lines (in vitro)	ERK1/JNK	Migration and invasion	[65]
	Neuroblastoma	SH-EP, SK-N-SH, and ZMR32 human cell lines (in vitro)	FoxO	Migration	[62]
Anti-tumorigenic action	Lung cancer	A549, H460, H1975, H157, and H838 human cell lines (in vitro)	p120 catenin	Cadherin-mediated invasion	[73]
	Pancreatic ductal adenocarcinoma (PDAC)	PANC-1 human cell line, PancO2 murine cell line, primary PDAC cells from PDX models (in vitro), and mice model (in vivo)	EGFR and TGF- β/p38/Smads	Cell growth	[66]
	Pancreatic ductal adenocarcinoma (PDAC)	PANC-1 human cell line, primary cell cultures (in vitro), and tissue biopsies from PDX model (ex vivo)	HIF-1a and AMPK	Cell growth	[67]
	Melanoma	A375 human cell line (in vitro)	Integrin-β1/FAK/vinculin	Migration	[69]
	Melanoma	B16F1 human cell line (in vitro) and mice model (in vivo)	Snail1	Metastasis and invasion	[66]
	Breast cancer	MCF-7/c and MDA-MB-231 human cell lines (in vitro)	CD44/Hyaluronan synthase and Integrin-α1 and -β1/FAK/ERK1/2/MAPK 42/44/Akt	EMT metastasis	[47]
